# Supplementary material for: Immunophenotypic Landscape and Prognosis of Diffuse Large B-Cell Lymphoma with MYC/BCL2 Double Expression: An Analysis of A Prospectively Immunoprofiled Cohort
Source: Cancers (Basel). 2020 Nov 9;12(11):3305. doi: 10.3390/cancers12113305 (PMC7697982; doi:10.3390/cancers12113305)
Supplement: Supplementary file 1 [file cancers-12-03305-s001.pdf]

Supplementary Materials:

Immunophenotypic Landscape and Prognosis of Diffuse Large B-Cell Lymphoma with MYC/BCL2 Double Expression: An Analysis of A Prospectively Immunoprofiled Cohort

Bogyeong Han, Sehui Kim, Jiwon Koh, Jeemin Yim, Cheol Lee, Dae Seog Heo, Tae Min Kim, Jin Ho Paik and Yoon Kyung Jeon

Table S1. Univariate survival analysis of OS and PFS according to clinicopathological parameters in DLBCL patients treated with R-CHOP.

Variables	PFS			OS		
	**HR**	**95% CI**	***P***	**HR**	**95% CI**	***P***
Age (year)	1.014	0.997–1.031	0.103	1.038	1.022–1.054	<0.001
Age > 60 years	1.436	0.926–2.229	0.106	2.208	1.469–3.320	<0.001
Sex (female)	0.894	0.578–1.382	0.613	0.662	0.449–0.976	0.037
Ann Arbor Stage III–IV	3.217	2.073–4.992	<0.001	3.964	2.686–5.849	<0.001
Presence of B symptoms	1.942	0.894–4.220	0.094	1.811	0.915–3.584	0.088
Presence of bulky disease	0.236	0.058–0.959	0.043	0.356	0.131–0.967	0.043
ECOG PS ≥2	2.286	1.218–4.290	0.01	3.956	2.536–6.170	<0.001
Elevated serum LDH	4.789	2.757–8.317	<0.001	7.593	4.314–13.364	<0.001
No. of extranodal sites ≥2	1.895	1.207–2.976	0.005	2.117	1.450–3.091	<0.001
Presence of BM involvement	1.684	0.916–3.093	0.093	3.504	2.234–5.494	<0.001
IPI ≥2	2.479	1.482–4.147	0.001	3.928	2.340–6.593	<0.001
BCL2 ≥ 70%	3.552	2.102–6.002	<0.001	2.627	1.716–4.022	<0.001
MYC ≥ 40%	1.883	1.209–2.934	0.005	2.244	1.518–3.317	<0.001
Ki-67 (%)	1.001	1.000–1.001	0.171	1.001	1.000–1.001	0.092
CD10 ≥ 30%	0.823	0.479–1.414	0.481	1.021	0.656–1.587	0.928
BCL6 ≥ 30%	0.742	0.474–1.160	0.19	0.856	0.580–1.264	0.435
MUM1 ≥ 30%	1.577	0.980–2.540	0.061	1.412	0.940–2.122	0.096
COO of non-GCB	1.508	0.925–2.458	0.099	1.354	0.891–2.059	0.156
MYC/BCL2 DE	3.332	2.135–5.201	<0.001	2.968	2.018–4.364	<0.001

Abbreviations: DLBCL, diffuse large B-cell lymphoma; PFS, progression-free survival; OS, overall survival; HR, hazard ratio; CI, confidence interval; ECOG PS, Eastern Cooperative Oncology Group Performance Status; LDH, lactate dehydrogenase; No., number; BM, bone marrow; IPI, International Prognostic Index; R-CHOP, rituximab plus cyclophosphamide, doxorubicin, vincristine, and prednisone; COO, cell-of-origin; non-GCB, non-germinal center B-cell-like; DE, double expression.

Table S2. Multivariate survival analysis of OS and PFS according to clinicopathological parameters in GCB-DLBCL patients treated with R-CHOP.

**Variables**	**PFS**			**OS**		
	**HR**	**95% CI**	***P***	**HR**	**95% CI**	***P***
Comparison with risk factors						
Ann Arbor Stage III-IV	6.007	2.031–17.767	0.001	3.707	1.213–11.324	0.021
ECOG PS ≥2	.	.	.	6.513	1.819–23.326	0.004
No. of extranodal sites ≥2	.	.	.	3.874	1.503–9.981	0.005
MYC/BCL2 DE status	11.089	4.048–30.379	<0.001	9.814	3.342-28.823	<0.001
Comparison with IPI						
IPI ≥2	6.08	2.059–17.952	0.001	8.093	3.246–20.173	<0.001
MYC/BCL2 DE status	11.17	4.083–30.556	<0.001	12.952	5.024–33.391	<0.001

Abbreviations: DLBCL, diffuse large B-cell lymphoma; PFS, progression-free survival; OS, overall survival; HR, hazard ratio; CI, confidence interval; ECOG PS, Eastern Cooperative Oncology Group Performance Status; No., number; IPI, International Prognostic Index; R-CHOP, rituximab, cyclophosphamide, doxorubicin, vincristine, and prednisone; GCB, germinal center B-cell-like; DE, double expression.

Table S3. Multivariate survival analysis of OS and PFS according to clinicopathological parameters in non-GCB-DLBCL patients treated with R-CHOP.

**Variables**	**PFS**			**OS**		
	**HR**	**95% CI**	***P***	**HR**	**95% CI**	***P***
Comparison with risk factors						
Age	.	.	.	2.062	1.039–4.091	0.038
Ann Arbor Stage III-IV	1.922	0.893–4.139	0.095	1.944	0.959–3.941	0.065
ECOG PS ≥2	.	.	.	2.255	1.268–4.011	0.006
Elevated serum LDH	4.360	1.909–9.957	<0.001	7.526	2.960–19.135	<0.001
MYC/BCL2 DE status	1.899	1.003–3.594	0.049	.	.	.
Comparison with IPI						
IPI ≥2	2.781	1.540–5.023	0.001	3.175	1.838–5.486	<0.001
MYC/BCL2 DE status	2.224	1.264–3.985	0.006	2.235	1.323–3.776	0.003

Abbreviations: DLBCL, diffuse large B-cell lymphoma; PFS, progression-free survival; OS, overall survival; HR, hazard ratio; CI, confidence interval; ECOG PS, Eastern Cooperative Oncology Group Performance Status; No., number; IPI, International Prognostic Index; R-CHOP, rituximab, cyclophosphamide, doxorubicin, vincristine, and prednisone; non-GCB, non-germinal center B-cell-like; DE, double expression.

Table S4. Univariate survival analysis of PFS and OS according to clinicopathological parameters in DE-DLBCL patients treated with R-CHOP.

Variables	PFS			OS		
	**HR**	**95% CI**	***P***	**HR**	**95% CI**	***P***
Age (year)	1.018	0.992–1.045	0.181	1.028	1.005–1.052	0.017
age >60 years	1.857	0.878–3.931	0.106	2.199	1.150–4.203	0.017
Female	1.007	0.532–1.906	0.983	0.803	0.458–1.407	0.442
Ann Arbor Stage III-IV	2.658	0.816–8.656	0.105	1.773	0.702–4.477	0.226
Presence of B symptoms	1.41	0.549–3.623	0.475	1.102	0.437–2.777	0.836
Presence of bulky disease	0.043	0.000–8.315	0.241	0.201	0.028–1.458	0.112
ECOG PS ≥2	1.248	0.509–3.057	0.628	2.124	1.085–4.157	0.028
Elevated serum LDH	3.251	1.416–7.464	0.005	4.757	2.122–10.664	<0.001
No. of extranodal sites ≥2	1.675	0.875–3.207	0.12	1.914	1.101–3.330	0.021
BM involvement	2.111	0.865–5.147	0.101	3.258	1.666–6.372	0.001
IPI ≥2	2.099	0.923–4.772	0.077	2.41	1.170–4.963	0.017
Ki-67 (%)	1.000	0.999–1.001	0.911	1.000	0.999–1.001	0.732
CD10 ≥30%	2.922	1.263–6.756	0.012	2.679	1.326–5.412	0.006

BCL6 ≥30%	0.747	0.387–1.443	0.385	0.656	0.372–1.159	0.147
MUM1 ≥30%	0.74	0.370–1.482	0.395	0.605	0.337–1.084	0.091
COO of non-GCB	0.631	0.275–1.448	0.277	0.503	0.255–0.989	0.046

Abbreviations: DE, double expression; DLBCL, diffuse large B-cell lymphoma; PFS, progression-free survival; OS, overall survival; HR, hazard ratio; CI, confidence interval; ECOG PS, Eastern Cooperative Oncology Group Performance Status; LDH, lactate dehydrogenase; No., number; BM, bone marrow; IPI, International Prognostic Index; R-CHOP, rituximab plus cyclophosphamide, doxorubicin, vincristine, and prednisone; COO, cell-of-origin; non-GCB, non-germinal center B-cell-like.

Table S5. Multivariate survival analysis of PFS and OS according to clinicopathological parameters in DE-DLBCL patients treated with R-CHOP.

Variables	PFS			OS		
	**HR**	**95% CI**	***P***	**HR**	**95% CI**	***P***
Comparison with risk factors						
Age	1.029	0.995–1.065	0.094	1.03	1.001–1.061	0.046
ECOG PS of ≥2	.	.	.	1.92	0.914–4.030	0.085
Elevated LDH	2.231	0.935–5.324	0.07	4.392	1.727–11.170	0.002
COO of non-GCB	.	.	.	0.361	0.154–0.845	0.019
Comparison with IPI						
IPI score of ≥2	1.809	0.792–4.133	0.16	2.897	1.325–6.335	0.008
COO of non-GCB	.	.	.	0.385	0.192–0.772	0.007

Abbreviations: DE, double expression; DLBCL, diffuse large B-cell lymphoma; PFS, progression-free survival; OS, overall survival; HR, hazard ratio; CI, confidence interval; ECOG PS, Eastern Cooperative Oncology Group Performance Status; LDH, lactate dehydrogenase; IPI, International Prognostic Index; R-CHOP, rituximab plus cyclophosphamide, doxorubicin, vincristine, and prednisone; COO, cell-of-origin; non-GCB, non-germinal center B-cell-like.

Table S6. Clinicopathological characteristics of patients in the validation set from SNUBH.

Variables*		DLBCL (n=260) No. (%)
Sex	male	160 (61.5)
	female	100 (38.5)
Age, years	median ± SD	65.50 ± 16.104
	mean ± SD	62.25 ± 16.104
Primary sites	nodal	102 (39.2)
	extranodal	158 (60.8)
Ann Arbor stage	1 or 2	101 (39.9)
	3 or 4	152 (60.1)
B symptoms	absent	69 (87.3)
	present	10 (12.7)
Bulky disease	absent	198 (77.3)
	present	58 (22.7)
ECOG PS	0 or 1	9 (47.4)
	2 or more	10 (52.6)
Serum LDH	normal	200 (76.9)
	elevated	60 (23.1)
No. of extranodal sites	0 or 1	196 (76.9)
	2 or more	59 (23.1)
BM involvement	absent	165 (80.1)
	present	41 (19.9)
IPI	0–1, low risk	65 (80.3)
	2, low-int risk	3 (3.7)
	3, high-int risk	5 (6.2)

	4–5, high risk	8 (9.9)
Tx regimen	R-CHOP	225 (93.4)
	others	16 (6.6)
Response to Tx	CR	136 (69.7)
	non-CR	59 (30.3)
BCL2	negative	72 (27.7)
	positive	188 (72.3)
MYC	negative	180 (69.2)
	positive	80 (30.8)
MYC/BCL2 DE status	Non-DE	200 (76.9)
	DE	60 (23.1)
CD10	negative	200 (76.9)
	positive	60 (23.1)
BCL6	negative	200 (76.9)
	positive	60 (23.1)
MUM1	negative	200 (77.5)
	positive	58 (22.5)
COO	GCB	82 (31.5)
	non-GCB	178 (68.5)

* Some variables have missing values. † Bulky disease was defined as tumor measured above 10 cm in the greatest dimension. Abbreviations: DLBCL, diffuse large B-cell lymphoma; ECOG PS, Eastern Cooperative Oncology Group Performance Status; LDH, lactate dehydrogenase; No., number; BM, bone marrow; IPI, International Prognostic Index; Int., intermediate; Tx, treatment; R-CHOP, rituximab, cyclophosphamide, doxorubicin, vincristine, and prednisone; CR, complete response; DE, double expression; COO, cell-of-origin; GCB, germinal center B-cell-like.

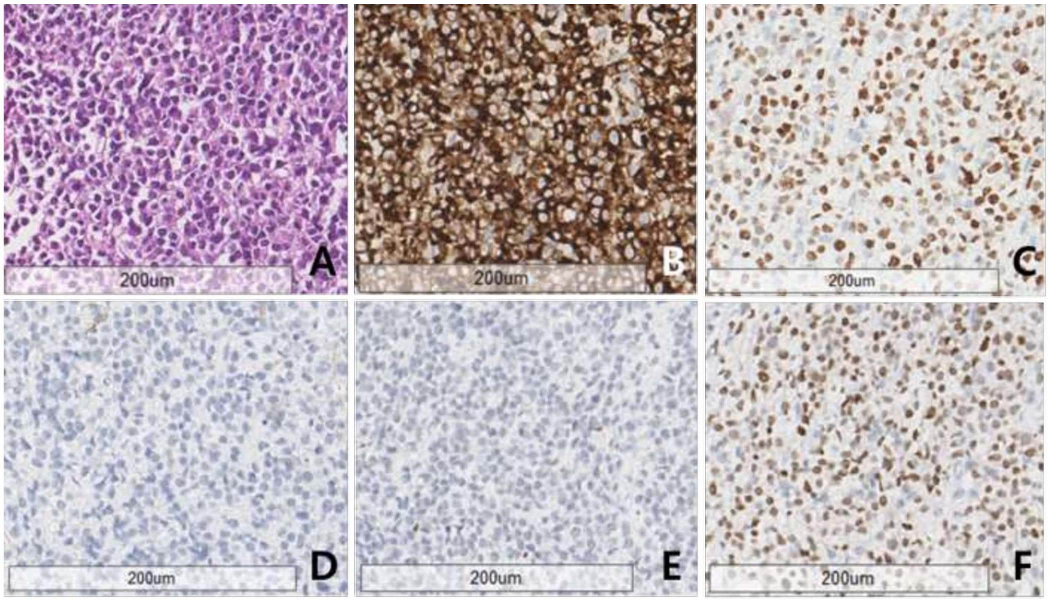

Figure S1. Representative pathological images of DE-DLBCL patient of non-GCB phenotype. Images of Hematoxylin & Eosin staining (**A**), BCL2 (**B**), MYC (**C**), CD10 (**D**), BCL6 (**E**) and MUM1 (**F**) immunohistochemical staining from a patients with DLBCL.

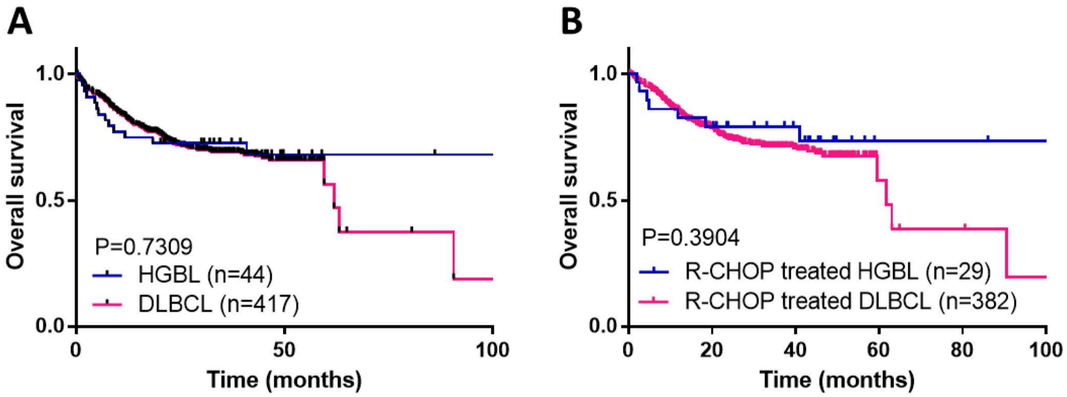

Figure S2. Overall survival of aggressive B cell lymphoma patients. Kaplan-Meier curve showing no difference in the overall survival (OS) of patients with DLBCLs and HGBLs in the whole cohort (**A**) or in the sub-cohort of patients treated with R-CHOP (**B**).

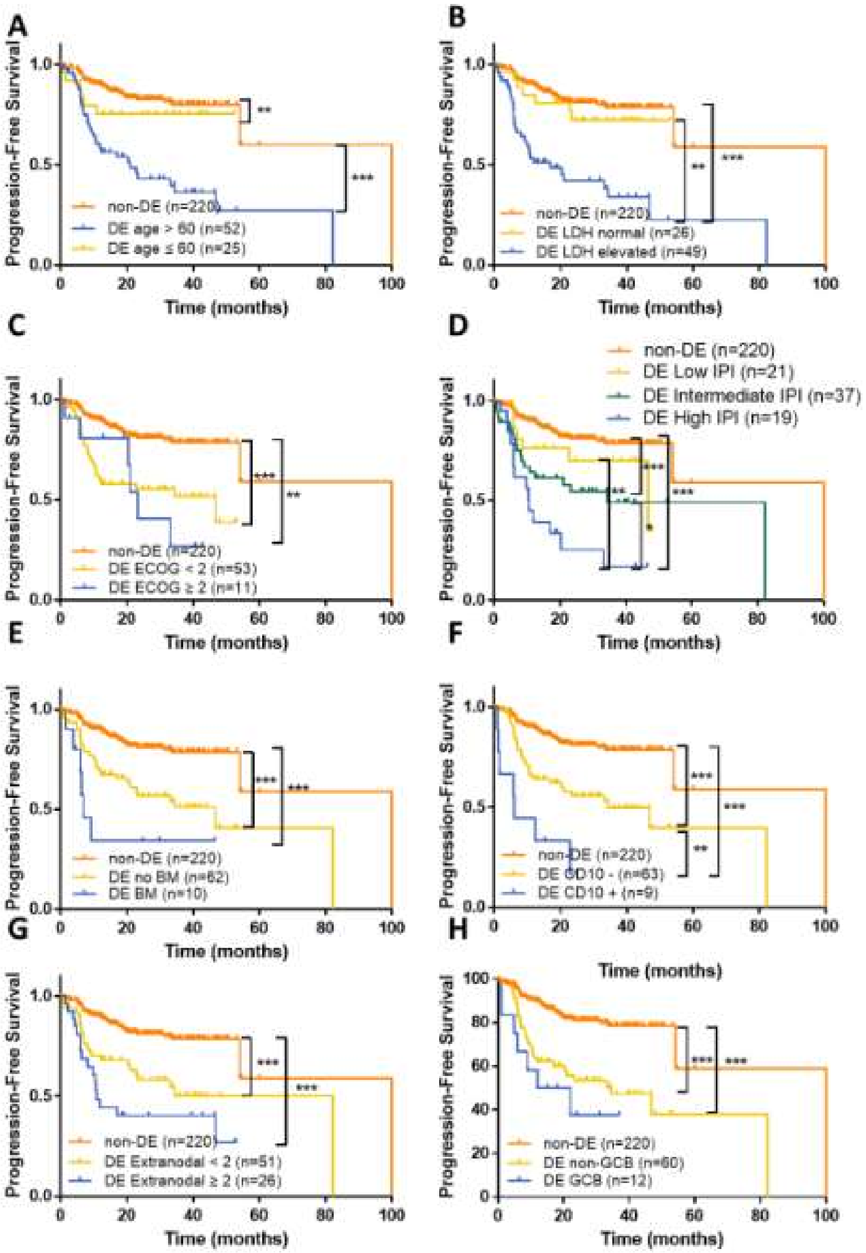

Figure S3. Progression-free survival of DLBCL patients treated with R-CHOP according to the DE status and clinicopathological parameters. PFS of DLBCL patients treated with R-CHOP according to the DE status and age (**A**), serum LDH levels (**B**), ECOG PS (**C**), IPI score (**D**), BM involvement (**E**), CD10 expression (**F**), number of extranodal sites (**G**), and COO (**H**).

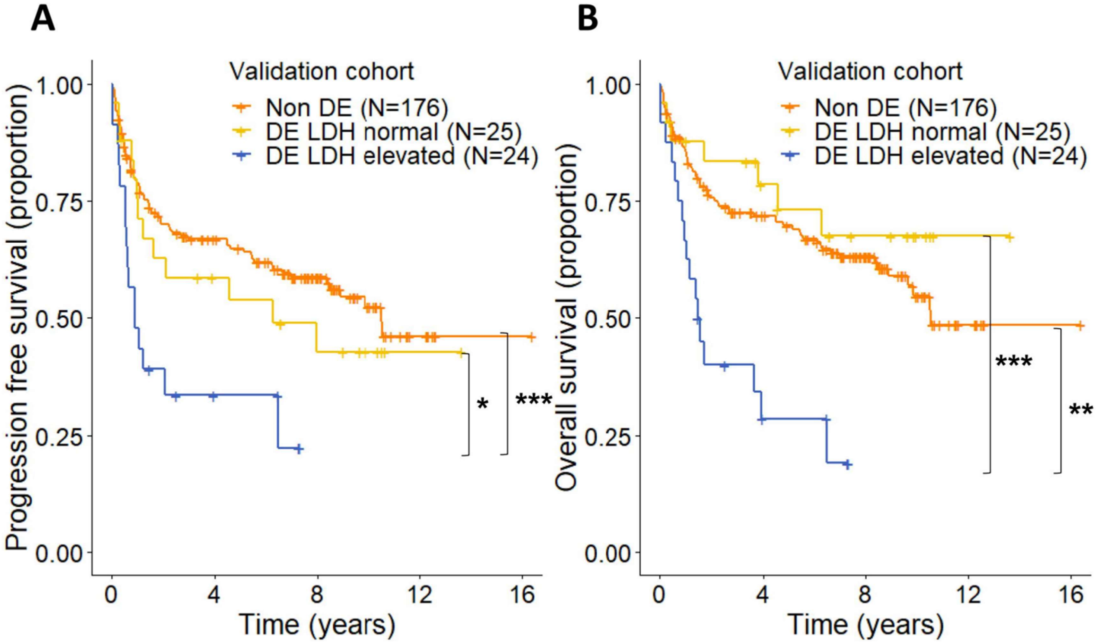

Figure S4. Survival of DLBCL patients according to the DE status and serum LDH levels in the publicly available validation set**.** (**A**) DE-DLBCL patients with elevated serum LDH levels exhibited significantly poor PFS than non-DE-DLBCL patients ($P < 0.001$) and DE-DLBCL patients with normal serum LDH levels ($P = 0.047$). No significant survival differences were observed between DE-DLBCL patients with normal serum LDH levels and non-DE-DLBCL patients ($P = 0.468$) (**B**) DE-DLBCL patients with elevated serum LDH levels exhibited poor OS than non-DE-DLBCL patients ($P < 0.001$) and DE-DLBCL patients with normal serum LDH levels ($P = 0.001$), whereas OS between DE-DLBCL patients with normal serum LDH levels and non-DE-DLBCL patients was similar ($P = 0.408$).

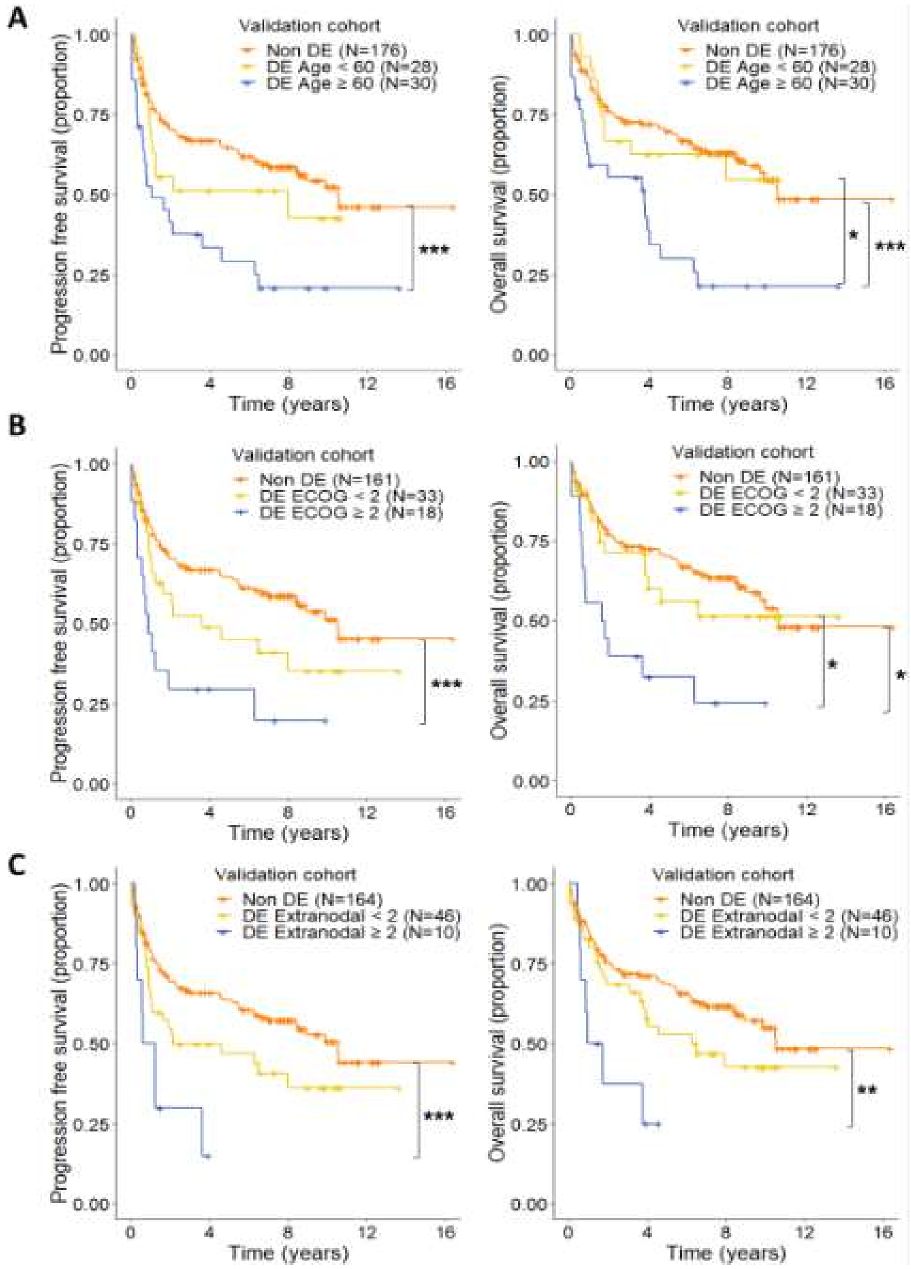

Figure S5. Survival of DLBCL patients according to the DE status and IPI-related variables in the publicly available validation set. Kaplan-Meier curves showing PFS and OS of DLBCL patients of publicly available validation set according to the DE status and age (A), patients' performance status (B), and number of involved extranodal sites (C).

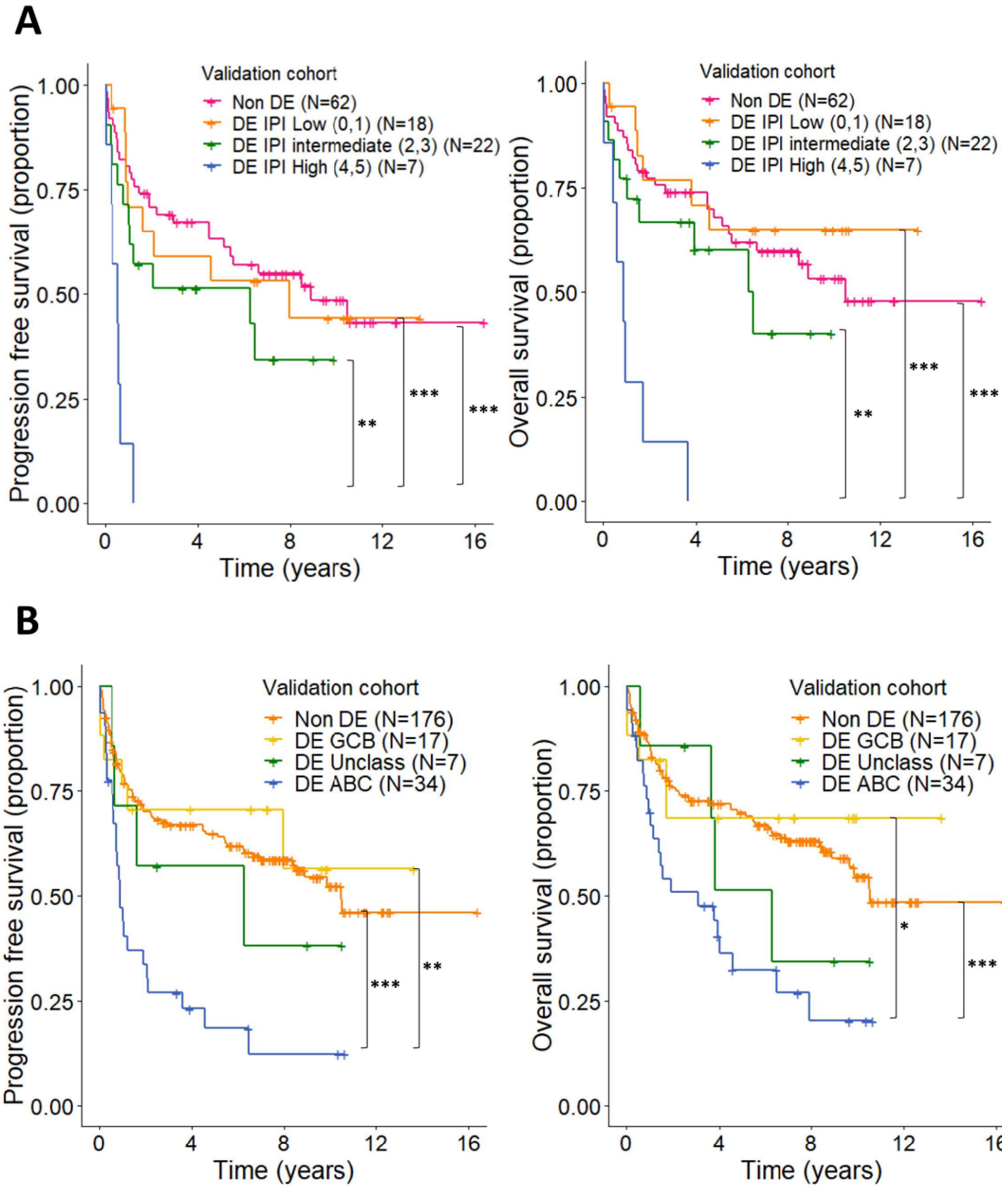

Figure S6. Survival of DLBCL patients according to the DE status and IPI and COO subgroups in publicly available validation set. Kaplan-Meier curves showing PFS and OS of DLBCL patients of publicly available validation set according to the DE status and IPI scores (**A**) and COO subgroups (**B**).

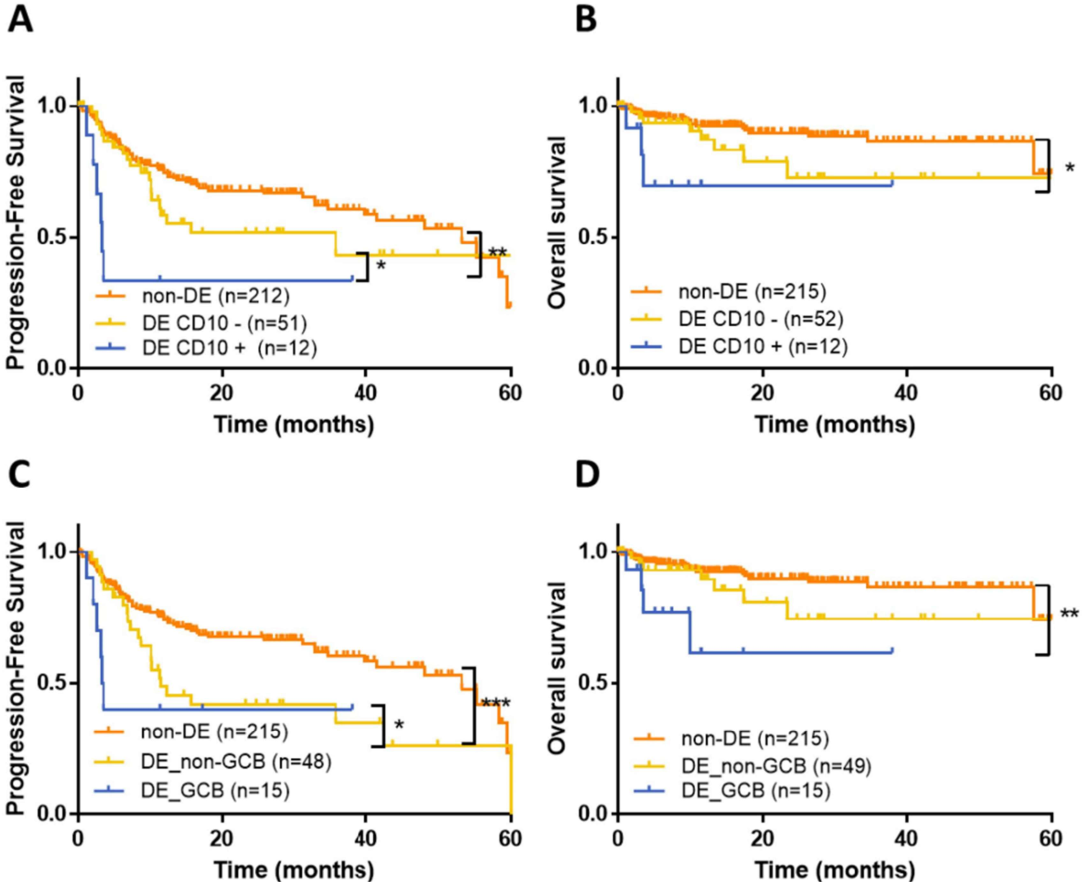

Figure S7. Survival of DLBCL patients according to the DE status and CD10 expression and COO in the validation set from SNUBH. Kaplan-Meier curves showing PFS and OS of DLBCL patients of validation set from SNUBH cohort according to the DE status and CD10 expression (**A**,**B**), and COO (**C**,**D**).
